# Results of a Randomized Controlled Trial Evaluating the Impact of Conversion to LCP Tacrolimus on Neurologic Toxicities in Liver Transplant Recipients

**DOI:** 10.1155/ijh/4374144

**Published:** 2025-10-06

**Authors:** Kelsey S. Coffman, Gonzalo J. Revuelta, Nathan DeTurk, Charles Palmer, Hannah Culpepper, Morgan Overstreet, James Fleming, Kathleen Terry, Neha Patel, John McGillicuddy, Santosh Nagaraju, Teresa C. Rice, David J. Taber

**Affiliations:** ^1^Department of Pharmacy Services, Medical University of South Carolina (MUSC), Charleston, South Carolina, USA; ^2^Department of Neurology, MUSC, Charleston, South Carolina, USA; ^3^Department of Surgery, MUSC, Charleston, South Carolina, USA; ^4^Department of Pharmacy, Ralph H Johnson VAMC, Charleston, South Carolina, USA

**Keywords:** liver transplantation, tacrolimus, tremor

## Abstract

**Background/Aims:**

Neurotoxicity is commonly seen in liver transplant (LT) patients receiving tacrolimus. We sought to determine the impact of LCP tacrolimus on neurologic toxicity in LT recipients.

**Methods:**

This single-center, semiblinded, parallel group randomized controlled trial compared neurotoxicity burden in LT patients receiving immediate-release (IR) tacrolimus versus LCP tacrolimus. Thirty LT recipients transplanted between January 2020 and February 2022 were enrolled between 15 and 364 days posttransplant and followed for 6 months postrandomization. The primary endpoint was change from baseline to 6 months in composite Patient Global Impression of Improvement (PGI-I) score. Select secondary endpoints included change in Fahn–Tolosa–Marin (FTM) Tremor Rating Scale, IMAB-Q10, SF-12, and Medical Symptom Validity Test (MSVT) scores.

**Results:**

No significant differences were seen in composite PGI scores, though all patients saw improvement in overall PGI scores (IR −5 [−13.5 to −0.25] vs. LCP −4 [−9.5 to −0.5], *p* = 0.78). Other tests examining neurotoxicities showed no difference between groups but an overall trend toward improvement in symptoms between baseline and end of study. One episode of moderate rejection (rejection activity index [RAI] score of 6) was reported in the LCP group, with no episodes in the IR group (*p* = 0.31). No graft loss or mortality occurred in either group.

**Conclusions:**

Our study showed LCP tacrolimus had similar rates of neurotoxicity in LT recipients compared to IR without increasing the risk of rejection, graft loss, or mortality; these results suggest LCP tacrolimus can be a safe alternative in LT recipients.

**Trial Registration:**

ClinicalTrials.gov identifier: NCT03823768

## 1. Introduction

Neurotoxicity is commonly reported in liver transplant (LT) recipients taking tacrolimus, with published rates of 15%–30% [1]. This spectrum of toxicities can range from mild (i.e., tremors and headaches) to severe (i.e., seizures and posterior reversible encephalopathy syndrome [PRES]). Neurotoxicity is a significant cause of morbidity after liver transplantation and the most common adverse effect (AE) leading to discontinuation of tacrolimus in abdominal transplant recipients [2]. Previous studies by Wiesener et al. and Haddad et al. have shown improved rejection rates in LT recipients taking immunosuppression regimens including tacrolimus compared to cyclosporine, highlighting the desire to maintain LT recipients on tacrolimus-based regimens [3, 4]. Although most literature evaluating tacrolimus-induced neurotoxicities in LT focuses on severe neurotoxicities, including seizures and PRES, evidence has shown milder neurotoxicities can persist and lead to significantly worse visuospatial/constructional ability, impaired cognitive function, and white brain matter changes [5]. In several chronic disease states, adverse drug events have been established as a major risk factor for medication nonadherence and are associated with significant healthcare utilization and costs [6, 7].

LCP tacrolimus (Envarsus) is a once-daily tacrolimus product associated with lower peak levels compared to immediate-release (IR) tacrolimus [8, 9]. Currently, LCP tacrolimus is approved for use in kidney transplant recipients, though use in other organ recipients has been published [8]. Alloway et al. previously reported LT recipients converted from IR to LCP tacrolimus at a conversion ratio of 0.7 had a similar *C*_min_ (trough) while also demonstrating significantly less variability in tacrolimus levels [10]. In an extension study of this group, the PK of LCP tacrolimus remained stable up to 26 weeks postconversion with no safety concerns up to 52 weeks after conversion [10]. In kidney transplant recipients experiencing clinically significant tremor, LCP tacrolimus has demonstrated significant improvement in tremors compared to IR [11]. Hand tremors exist with high incidence in LT recipients, worsening after administration of cyclosporine or tacrolimus [12]. As tremors can contribute to reduced quality of life (QoL), the possible benefit of LCP tacrolimus in LT is an area of interest.

The impact of LCP tacrolimus on neurotoxicities has not been well established in LT recipients. Previous literature in kidney transplant recipients showing significant improvement in select neurotoxic AEs, such as tremors, highlights potential benefit for use of LCP tacrolimus in LT recipients. The objective of this study was to evaluate differences in neurotoxicity burden in LT recipients receiving once-daily LCP tacrolimus compared to twice-daily IR tacrolimus.

## 2. Materials and Methods

This single-center, semiblinded, randomized comparative trial included LT recipients transplanted between January 2020 and February 2022. Patients were screened for enrollment if they received a liver or combined liver–kidney transplant within 15–364 days, were 18 years or older, and could provide written informed consent. Exclusion criteria included human immunodeficiency virus (HIV) positivity, pregnancy in the patient or spouse, inability to take medications by mouth, use of other investigational products within 30, and moderate acute cellular rejection (defined rejection activity index [RAI] of 5 or greater) episodes within a month. Patients with conditions causing tremor, those taking tremor-inducing medications or dopamine-blocking agents, and those with a condition or disorder that may adversely affect the study outcome or safety of the subject were not eligible for inclusion. The protocol for this research project has been approved by a suitably constituted Ethics Committee of the institution, and it conforms to the provisions of the Declaration of Helsinki (Approval Number Pro00083855; see supporting information (available here)). All informed consent was obtained from the subject(s) and/or guardian(s) by a research team member that completed Human Subjects Protection training.

A total of 30 patients were enrolled and randomized to control (receiving IR tacrolimus) or intervention (receiving LCP tacrolimus). Three random permuted blocks of 10 with equal arm allocation ratios were utilized in REDCap (Research Electronic Data Capture) to ensure 1:1 randomization within each block and the overall cohort. Subjects in each group received current standard of care immunosuppression prior to randomization. Standard of care regimens included any IR tacrolimus formulation dosed twice daily (goal 12-h trough: 3–10 ng/mL) ± adjunctive agent ± prednisone taper. Center protocol involved discontinuation of antimetabolite at 2 months posttransplant and prednisone at 3 months posttransplant in patients without autoimmune etiology of liver disease or history of severe rejection episode. Those in the control arm continued the standard regimen while those randomized to intervention converted from IR to LCP tacrolimus (goal 24-h trough: 3–10 ng/mL) at a dose conversion of 1-mg IR: 0.8 mg LCP, with the same adjunctive agents as the control group for a total of 6 months. Those randomized to the intervention arm were supplied medication free of charge from Veloxis Pharmaceuticals (Cary, North Carolina, United States); medication was not supplied for those in the control arm. Due to feasibility issues associated with blinding twice-a-day (IR) versus once-a-day (LCP) regimens, subjects were not blinded to their treatment arm. Patient baseline evaluations could occur up to 14 days prior to or 48 h after randomization. End of study evaluations occurred at least 6 months after randomization.

The primary outcome was the change in neurotoxicity burden, defined as the difference in composite Patient Global Impression of Improvement (PGI-I) score from baseline to 6 months, comparing those maintained on IR tacrolimus versus LCP tacrolimus. Other outcomes evaluated at baseline and 6-month follow-up included the change in Fahn–Tolosa–Marin (FTM) Tremor Rating Scale (TRS) score assessing tremor, the Identification of Medication Adherence Barriers Questionnaire (IMAB-Q10) score assessing adherence, the 12-Item Short Form Survey (SF-12) score assessing QoL, and the Medical Symptom Validity Test (MSVT) score assessing memory. TRS assessments at baseline and the end of study were video recorded and evaluated by a neurologist blinded to treatment arms.

Safety outcomes, evaluated at baseline, 3 months, and end of study, included mortality, graft loss, biopsy proven acute rejection (BPAR), hospitalizations, emergency department (ED) visits, and AEs. AEs were defined as any untoward medical occurrence after written informed consent. Severity of AEs was graded utilizing NCI Common Terminology Criteria for Adverse Events Version 4.03 (CTCAE). Laboratory values were assessed at baseline, Days 7 and 14, and monthly until 6 months. Pertinent laboratory values included comprehensive metabolic panels, complete blood counts, and tacrolimus trough levels. The four-variable abbreviated Modification of Diet in Renal Disease (MDRD) equation was utilized to assess renal function [13].

A sample size of 30 patients was calculated as necessary to demonstrate a mean difference in neurologic toxicity burden, using the PGI-I rating of 20 ± 15 with greater than 80% power using a two-tailed *t*-test with an alpha set at 0.05. Based on previously reported neurotoxicity rates, a mean score of 45 was expected at baseline. Using independent-samples *t*-test comparing two groups with a two-sided significance level of 0.05, a sample size of 14 subjects per group would be required to obtain a power of at least 0.8 if the two groups have a mean of 25 ± 15 neurologic toxicity burden in the LCP cohort at the conclusion of the study and a mean of 45 ± 20 neurologic toxicity burden at baseline and follow-up in the tacrolimus IR group. Estimating a 10% dropout rate, we proposed a sample size of 15 subjects per group. This sample size would provide adequate power to show a statistical and clinical difference in the neurotoxicity burden between the two groups.

All data was stored in REDCap electronic data capture tools [14, 15]. The OPTN/UNOS STAR file, manually abstracted data, and EMR abstracted data were then merged into Microsoft Excel CSV files (Microsoft Corp, Redmond, Washington). Data was evaluated under the intention-to-treat principle. Proportions (%), means, and standard deviations (SDs) were utilized for reporting parametric data, including baseline characteristics, laboratory values, and safety outcomes. These results were analyzed using chi-square for categorical data and paired *t*-tests for continuous data. Medians and 25%–75% interquartile ranges (IQRs) were utilized to report nonparametric data, including scores from PGI-I, TRS, IMAB-Q10, SF-12, and MSVT; independent-samples Mann–Whitney *U* tests were used for analysis of these results. SPSS v24.0 (IBM Corp, Armonk, New York) was utilized to conduct statistical analyses.

## 3. Results

A total of 30 LT recipients were enrolled between January 2020 and February 2022, with 15 subjects randomized to each arm. One patient (6.7%) withdrew in the control group. A total of 3 subjects (20%) in the IR group and 4 (26.7%) in the LCP arm were lost to follow-up or refused end of study assessments (see supporting information). Baseline characteristics are displayed in Table 1. Most participants were female, Caucasian, and in their early 50s. The most common indications for transplant included alcohol-associated cirrhosis (IR 46.7% vs. LCP 53.3%, *p* = 0.72) and nonalcoholic steatohepatitis (IR 26.7% vs. LCP 46.7%, *p* = 0.26). No other significant differences in baseline characteristics were identified between groups. Donor characteristics were similar in both groups except for the increased incidence of hypertension in the IR group (IR 40% vs. LCP 6.7%, *p* = 0.031).

Overall PGI scores were similar between control and intervention groups at baseline and end of study, as well as in each symptom category (see Table 2). All patients saw improvement in overall PGI scores at the end of the study period (IR −5 [−13.5 to −0.25] vs. LCP −4 [−9.5 to −0.5], *p* = 0.78). No differences were seen between study groups in change from baseline to end of study, either overall, or in any symptom category. Tremor evaluation via TRS scoring was similar at baseline and end of study in each treatment arm (see Table 3); median TRS scores were low in both groups. Though not statistically significant, the difference in median TRS scoring from baseline to 6-month follow-up was numerically higher in the IR group (−4.9 [−7.7 to 1.8]) compared to the LCP group (−1.2 [−3.9 to 1.3]) (*p* = 0.22). Medication adherence, evaluated by IMAB-Q10 (see Table 4), was similar at baseline and end of study, aside from more patients in the IR group having baseline score > 20 (IR 46.7% vs. LCP 13.3%, *p* = 0.046). Scores > 20 are associated with increased rates of self-reported nonadherence. Improvement in median IMAB scores was seen in each group (IR −5 vs. LCP −2, *p* = 0.55). Median SF-12 scores, evaluating patient-reported QoL, were similar between groups at baseline (IR 48.2% [37.5–59] vs. LCP 46.4 [35.7–53.6], *p* = 0.51) and end of study (IR 57.1% [44.6–62.9] vs. LCP 58.9% [41.1–67], *p* = 0.91), with no significant difference between IR and LCP groups in improvement from baseline seen at 6-month follow-up (IR median 6.25% [−4.46 to 25.9] vs. LCP 10.7% [−3.57 to 15.2], *p* = 1). Median MSVT scores evaluating memory were similar between groups at baseline and end of study in each group (see Table 5). Differences in MSVT scores were not significantly different in any category between beginning and end of study timeframes. MSVT scores of < 90, indicating impairment in memory, were similar between groups at baseline (IR 6.7% vs. LCP 6.7%, *p* = 1) and end of study (IR 0 vs. 0, *p* = 1).

**Figure 1:**
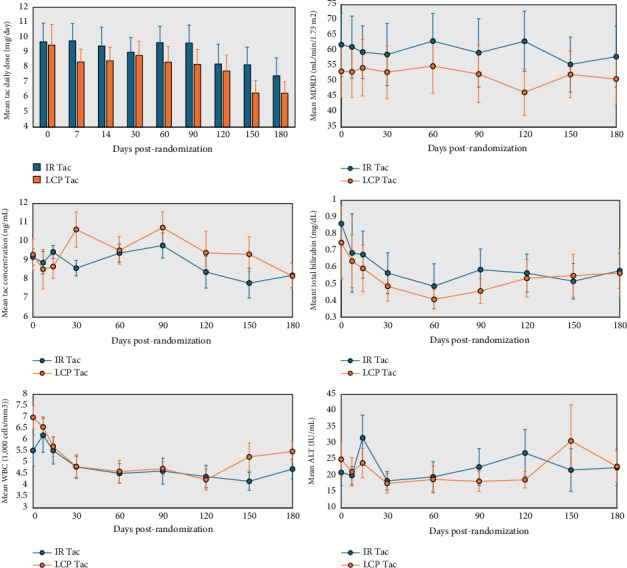
Pertinent clinical outcomes and laboratory values.

Immunosuppression trends were similar between groups. No significant differences were seen in tacrolimus doses (IR 9.7 ± 4.8 vs. LCP 9.5 ± 5.4, *p* = 0.9) or trough levels (IR 9.2 ± 1.7 vs. LCP 9.3 ± 3.2, *p* = 0.89). Tacrolimus dosing and trough level trends were similar at baseline and throughout treatment, though trough levels trended toward higher and median daily dose trended toward lower in the LCP arm (see Figure 1). Tacrolimus was held in two subjects (13.3%) in the control and three (20%) subjects in the intervention groups, respectively (*p* = 0.62); indications for holding included supratherapeutic trough levels (*n* = 2), seizure (*n* = 1), gastrointestinal (GI) symptoms (*n* = 1), and intentional nonadherence (*n* = 1). One patient in each arm discontinued tacrolimus during the follow-up period for drug-induced seizure (IR) and GI symptoms (LCP). The mean mycophenolate (MMF) dose at conversion was 1146 mg (±331) in the IR group versus 1178 mg (±372) in the LCP group (*p* = 0.82). Mycophenolate was held in four recipients (26.7%) in the control arm and one recipient (6.7%) in the intervention arm (*p* = 0.14) during the study period for leukopenia (*n* = 2), diarrhea (*n* = 1), COVID-19 infection (*n* = 1), and intentional nonadherence (*n* = 1). For patients on mycophenolate at the time of conversion (IR *n* = 10, LCP *n* = 14), mycophenolate was discontinued during the study period in four (40%) patients in the IR group and two patients (14.3%) in the LCP group. Mycophenolate doses for patients who remained on mycophenolate for the follow-up period were similar between groups at all time points. Prednisone doses at conversion (IR 11 ± 5.1 mg vs. LCP 11.5 ± 3.8 mg, *p* = 0.76) and discontinuation rates per protocol (IR 33.3% vs. LCP 40%, *p* = 0.71) were similar.

The most common adverse events reported included neurologic symptoms, GI symptoms, and pain; no significant differences were seen between groups (see Table 6). No graft loss or mortality was reported in either group during the follow-up period. One rejection episode with RAI of at least 5 occurred during the study period in the LCP group in the setting of multiple subtherapeutic FK levels, whereas no rejection episodes were seen in the IR group (*p* = 0.31). The single rejection episode responded to treatment with high-dose corticosteroids. Hospitalizations occurred more commonly in the control group (IR 6 episodes vs. LCP 2 episodes), though this was not statistically significant (*p* = 0.1). A similar number of ED visits were seen between groups (IR 3 vs. LCP 1, *p* = 0.28). Liver function test results had no significant differences between groups at any time points; trends of total bilirubin and alanine transaminase can be seen in Figure 1. White blood cell count results varied throughout the study period as seen in Figure 1, though there were not any significant differences between groups. Potassium levels > 6 mEq/L occurred in two (13.3%) intervention subjects and no control subjects (*p* = 0.14). Renal function, as estimated by the MDRD equation, did not differ between groups at any time point, though there was a trend toward lower eGFR in the LCP tacrolimus group at baseline and throughout the follow-up period, as demonstrated in Figure 1.

## 4. Discussion

The high rates of neurotoxicity associated with the use of IR tacrolimus in LT recipients highlight a desire to evaluate alternative immunosuppressive options that are effective while reducing these side effects. LCP tacrolimus has previously been shown to reduce tremor burden in renal transplant recipients who experienced significant tremor burden on IR tacrolimus [11]. As LCP tacrolimus is not currently approved for utilization in LT recipients, data evaluating neurologic outcomes of LCP in LT patients is limited. Our study found no difference between IR and LCP tacrolimus in neurologic AE burden, including tremor, memory, and overall QoL. These results were likely limited due to high rates of loss to follow-up secondary to the COVID-19 pandemic. Additionally, we chose all comers to include within this study, where targeting an enriched population of patients with current neurologic toxicity may have been able to better identify changes in toxicity between the two treatment approaches. Despite no difference seen in symptom burden, LCP tacrolimus was not associated with worse rejection, graft, or mortality rates; thus, utilization of LCP tacrolimus did not increase the risk of graft complications in our cohort. These findings add to the limited data available regarding the use of LCP tacrolimus in LT recipients. This is the first study, to our knowledge, to evaluate the impact of LCP tacrolimus on neurotoxicity burden in LT recipients.

As LCP tacrolimus is associated with reduced peak whole blood concentrations and improved exposure compared to IR, there is theoretical benefit to reduction of peak-associated neurologic AEs in LT recipients [8, 10]. As mentioned previously, this has been shown in renal transplant recipients but has not been extensively evaluated in LT^11^. Choi et al. evaluated outcomes of 25 LT recipients converted from IR to LCP tacrolimus. Most patients were converted from IR due to fluctuating tacrolimus levels or tremors. Of those converted for tremors, 88% reported significant improvement in tremor burden [16]. These results aligned with those previously reported by Langone et al. in renal transplant recipients [11]. DuBay et al. previously published results of their multicenter, Phase II, randomized controlled trial comparing de novo IR and LCP tacrolimus in 59 LT recipients. Tremor rates were not statistically significant between groups, though there was a trend toward lower rates associated with LCP (LCP 27.6% vs. IR 34.5%) [17]. Though our results did not find a difference between tacrolimus formulations, there was an improvement in tremor burden in those receiving LCP tacrolimus, as shown by median difference of −1.2 in TRS scores from baseline to end of study.

In addition to the impact on tremor burden in LT recipients, the efficacy of LCP tacrolimus is another important area to evaluate. Bilbao et al. compared rates of treatment failure, defined as the presence of BPAR, loss to follow-up, death, or graft failure, in LT recipients taking de novo LCP tacrolimus to those taking prolonged-release (PR) tacrolimus. Of the 156 patients analyzed, 26.9% experienced treatment failure, with no significant difference between treatment arms (LCP 30.5% vs. PR 23%, *p* = 0.29), suggesting similar efficacy between LCP and PR tacrolimus products [18]. DuBay et al. found similar rates of BPAR between IR (17.2%) and LCP (24.1%) groups [17]. The rejection rates observed in our population were significantly lower than those seen in this study, potentially secondary to the longer time to steady state associated with LCP tacrolimus, particularly when utilized de novo, and our reporting of only moderate (RAI 5+) rejections. Only one rejection episode was reported by Choi et al. in their LCP cohort, which was similar to the rejection rate seen in our LCP cohort (6.7%) [16]. Overall, our results support previous findings that LCP tacrolimus does not increase the risk of rejection or graft loss.

Neurotoxicities, in addition to other AEs, can significantly impact patient QoL. Overall QoL assessments were completed by Langone et al. in kidney transplant recipients and found significant improvements in Quality of Life in Essential Tremor Questionnaire (QUEST) scores after conversion from IR to LCP (mean change from baseline −7.4, *p* < 0.0001) [11]. The improvement in these results was predominantly driven by changes in physical, psychosocial, and work/financial areas, likely due to a reduction in neurotoxicity burden [11]. Though our study did not find a difference between groups in SF-12 scores, assessing QoL, improvement was seen in SF-12 scores in both IR and LCP groups from baseline to end of study. Improvement seen in QoL seen in the LCP group may be secondary to many factors, including reduced neurotoxicity and other AE burden, reduced medication administration associated with a once-daily tacrolimus regimen, and return to normal activities when further out from transplant. Another AE that can significantly impact patient QoL is nephrotoxicity due to potential need for renal replacement therapy, additional medications for management, and the addition of another specialist. DuBay et al. did not find any difference in eGFR between IR and LCP groups during their study follow-up period [17]. Similarly, no significant difference in eGFR or serum creatinine was observed by Choi et al. after conversion from IR to LCP tacrolimus [16]. These results align with our findings of no significant differences between IR and LCP groups, though the LCP group did trend toward having reduced renal function, which could be due in part to lower renal function at the time of conversion. The relatively short-term follow-up of the study may have impacted these results. Thus, long-term follow-up is necessary to better assess the risk of chronic nephrotoxicity and its impact on QoL.

There are several limitations of our study to note. Lack of follow-up limited overall data to be examined. The COVID-19 pandemic led to many in-person appointments being cancelled and moved to virtual visits; this reduced opportunities to reach patients and complete end of study assessments. Additionally, as LCP tacrolimus is not approved in LT recipients, providers managing and adjusting tacrolimus doses were not as familiar with the PK associated with LCP. This likely contributed to higher tacrolimus levels being maintained in intervention patients and could have contributed to the trend toward reduced eGFR in this group. Additionally, patients were able to be converted between 15 and 364 days posttransplant; thus, those converted closer to transplant may not have been stabilized on a dose of IR tacrolimus compared to those further out from transplant. AEs were identified based on chart documentation, which could have led to underreporting of patient-reported symptoms. Finally, we chose a general LT population instead of selecting an enriched population of patients already experiencing neurologic toxicities, which may have mitigated the different treatment methods. However, this would have substantially slowed the enrollment of the study, especially in the setting of the COVID-19 pandemic.

## 5. Conclusions

LCP tacrolimus was associated with similar change in neurotoxicity burden from baseline to 6-month follow-up in LT recipients compared to IR tacrolimus without increased rates of rejection, graft loss, rejection, or serious AEs. Small sample size and loss to follow-up likely contributed to a lack of difference in neurotoxicity burden seen with LCP, as has been shown in kidney transplant recipients. Future studies with larger sample sizes are needed to further evaluate the impact of LCP on neurologic AE burden in LT recipients.

## Figures and Tables

**Table 1:** Baseline characteristics.

	**IR (** **n** = 15**)**	**LCP (** **n** = 15**)**	**p** **value**
Age, mean (±SD)	51.3 (12.9)	51.8 (8.6)	0.9
Male sex, *n* (%)	6 (40)	2 (13.3)	0.099
African American, *n* (%)	1 (6.7)	2 (13.3)	0.54
Hispanic, *n* (%)	1 (6.7)	1 (6.7)	1
Private insurance, *n* (%)	10 (66.7)	11 (73.3)	0.69
Cytomegalovirus high risk (donor IgG+/recipient IgG−), *n* (%)	3 (20)	7 (46.7)	0.36
*Indication for transplant*			
Hepatitis C, *n* (%)	0	1 (6.7)	0.31
Alcohol-associated, *n* (%)	7 (46.7)	8 (53.3)	0.72
Nonalcoholic steatohepatitis, *n* (%)	4 (26.7)	7 (46.7)	0.26
Cryptogenic, *n* (%)	0	1 (6.7)	0.31
Hepatocellular carcinoma, *n* (%)	1 (6.7)	2 (13.3)	0.54
Autoimmune, *n* (%)	0	1 (6.7)	0.31
Primary biliary cholangitis, *n* (%)	0	1 (6.7)	0.31
Primary sclerosing cholangitis, *n* (%)	2 (13.3)	0	0.14
Fulminant, *n* (%)	0	1 (6.7)	0.31
Hilar cholangiocarcinoma, *n* (%)	1 (6.7)	0	0.31
Alpha-1 antitrypsin, *n* (%)	0	1 (6.7)	0.31
*Donor characteristics*			
Age, mean (±SD)	48.5 (15)	40.3 (10.6)	0.063
Donation after cardiac death, *n* (%)	2 (13.3)	1 (6.7)	0.54
Female sex, *n* (%)	6 (40)	3 (20)	0.23
African American, *n* (%)	3 (20)	1 (6.7)	0.28
CDC high risk, *n* (%)	3 (20)	4 (26.7)	0.67
Hepatitis B virus surface antibody positive, *n* (%)	2 (13.3)	0	0.143
Hepatitis C virus core antibody positive, *n* (%)	3 (20)	2 (13.3)	0.63
Hypertension, *n* (%)	6 (40)	1 (6.7)	0.031

**Table 2:** Patient Global Impression (PGI) Scale results.

	**IR**	**LCP**	**p** **value**
*Overall score*			
Baseline, median (IQR)	18 (14–21)	17 (16–20)	0.94
End of study, median (IQR) IR *n* = 10, LCP *n* = 9	11 (8–15.25)	12 (6.5–15)	0.84
Difference, median (IQR)	−5 (−13.5 to −0.25)	−4 (−9.5 to −0.5)	0.78
*Tremor*			
Baseline, median (IQR)	5 (3–6)	4 (3–5)	0.19
End of study, median (IQR)	2.5 (1.75–4)	3 (1.5–3.5)	0.84
Difference, median (IQR)	−2 (−5 to 0)	−1 (−2 to 0)	0.45
Improved by 1+ points, *n* (%)	6 (60)	7 (77.8)	0.41
*Headaches*			
Baseline, median (IQR)	4 (4–5)	4 (4–5)	0.74
End of study, median (IQR)	3.5 (1.75–4)	4 (1.5–4)	0.84
Difference, median (IQR)	−1 (−3 to −0.75)	−1 (−3.5 to 0)	0.72
Improved by 1+ points, *n* (%)	5 (50)	4 (44.4)	0.81
*Insomnia*			
Baseline, median (IQR)	3 (3–6)	4 (2–5)	0.87
End of study, median (IQR)	2 (1.75–4)	2 (1–4)	0.72
Difference, median (IQR)	−1 (−3 to 0.25)	−1 (−2.5 to 0.5)	1
Improved by 1+ points, *n* (%)	7 (70)	7 (77.8)	0.7
*Paresthesia*			
Baseline, median (IQR)	4 (3–6)	4 (4–5)	0.74
End of study, median (IQR)	3 (1.75–4)	2 (1.5–3.5)	0.5
Difference, median (IQR)	−1 (−4 to 0.25)	−1 (2.5 to −0.5)	0.97
Improved by 1+ points, *n* (%)	5 (50)	7 (77.8)	0.21

**Table 3:** Fahn–Tolosa–Marín (TRS) Scale results.

	**IR**	**LCP**	**p** **value**
Baseline, median % (IQR)	6.9 (4.2–14.6)	5.6 (0.7–8.6)	0.14
End of study, median % (IQR) IR *n* = 10, LCP *n* = 9	5.6 (1.6–10.3)	5.6 (2.9–7.3)	0.81
Difference, median % (IQR)	−4.9 (−7.7 to 1.8)	−1.2 (−3.9 to 1.3)	0.22

**Table 4:** Identification of Medication Adherence Barriers Questionnaire (IMAB-Q) results.

	**IR**	**LCP**	**p** **value**
Baseline, median (IQR)	18 (15–23)	15 (12–17)	0.089
End of study, median (IQR) IR *n* = 10, LCP *n* = 9	15.5 (11–19.25)	14 (12–18)	0.84
Difference, median (IQR)	−5 (−9.25 to 2.25)	−2 (−4.5 to 1)	0.55
Improvement of 2+ points, *n* (%)	6 (60)	5 (55.5)	0.85
Baseline score > 20, *n* (%)	7 (46.7)	2 (13.3)	0.046
End of study score > 20, *n* (%)	2 (20)	1 (11.1)	0.6

**Table 5:** Medical Symptom Validity Test (MSVT) results.

	**IR**	**LCP**	**p** **value**
*Immediate recognition*			
Baseline, median % (IQR)	100 (100–100)	100 (100–100)	0.81
End of study, median % (IQR) IR *n* = 10, LCP *n* = 9	100 (100–100)	100 (100–100)	0.97
Difference, median % (IQR)	0 (0–2.5)	0 (0–0)	0.84
*Delayed recognition*			
Baseline, median % (IQR)	100 (95–100)	100 (95–100)	0.78
End of study, median % (IQR)	100 (100–100)	100 (100–100)	0.66
Difference, median % (IQR)	0 (0–1.25)	0 (0–2.5)	0.72
*Consistency*			
Baseline, median % (IQR)	100 (95–100)	100 (95–100)	0.6
End of study, median % (IQR)	100 (98.8–100)	100 (95–100)	0.66
Difference, median % (IQR)	0 (−1.25 to 1.25)	0 (0–0)	0.72
*Paired associates*			
Baseline, median % (IQR)	100 (100–100)	100 (100–100)	0.54
End of study, median % (IQR)	100 (100–100)	100 (100–100)	1
Difference, median % (IQR)	0 (0–0)	0 (0–0)	1
*Free recall*			
Baseline, median % (IQR)	70 (60–80)	80 (70–90)	0.29
End of study, median % (IQR)	85 (70–90)	70 (70–85)	0.5
Difference, median % (IQR)	0 (−2.5 to 10)	0 (−12.5 to 5)	0.24
*Immediate recognition, delayed recall, consistency composite*			
Baseline, median % (IQR)	100 (96.7–100)	100 (96.7–100)	0.68
End of study, median % (IQR)	100 (99.6–100)	100 (96.7–100)	0.66

**Table 6:** Safety.

	**IR**	**LCP**	**p** **value**
Hospitalizations, *n*	6	2	0.1
ED visit, *n*	3	1	0.28
Neurologic symptoms, *n* (%)	7 (46.6)	7 (46.6)	1
Gastrointestinal symptoms, *n* (%)	4 (26.7)	5 (33.3)	0.69
Pain, *n* (%)	3 (20)	5 (33.3)	0.41
Potassium > 6 mEq/L, *n* (%)	0	2 (13.3)	0.14
White blood cell < 3000/mm^3^, *n* (%)	12 (80)	8 (53.5)	0.12
White blood cell < 2000/mm^3^, *n* (%)	7 (46.7)	5 (33.3)	0.46

## Data Availability

The data that support the findings of this study are available from the corresponding author upon reasonable request.

## Supporting Information

Supporting Information Additional supporting information can be found online in the Supporting Information section. Information regarding the data collection schedule, the CONSORT flow chart, and CONSORT checklist for randomized trials can be found in the supporting information.
